# P55 Work ready champions for young people: the role of occupational therapy in addressing the pre-vocational and early-employment needs of young people with long term health conditions

**DOI:** 10.1093/rap/rkac067.055

**Published:** 2022-09-28

**Authors:** Claire Pidgeon, Janine Hackett, Laura Lunt, Albert Farre, Janet McDonagh

**Affiliations:** Birmingham Women's and Children's NHS Foundation Trust, Birmingham, United Kingdom; Queen Margaret University, Edinburgh, United Kingdom; Centre for MSK Research, Manchester, United Kingdom; NIHR Biomedical Research Centre, Mancheter, United Kingdom; University of Dundee, Dundee, United Kingdom; Versus Arthritis Centre for Epidemiology, University of Manchester, Manchester, United Kingdom; NIHR Biomedical Research Centre, Manchester, United Kingdom; Royal Manchester Children's Hospital, Manchester, United Kingdom

## Abstract

**Introduction/Background:**

Developing a vocational identity is an important part of adolescent development and vocational morbidities have been recognized in young people with long term health conditions including rheumatic disease. Challenges in educational settings and/or workplaces have been reported by young people. Young people have also identified this area to be a research priority. Occupational Therapy (OT) is well placed to assume a lead role in this area, as it is the only health profession that has occupation at the core of its philosophy and practice.

**Description/Method:**

The aim of this study was to explore the pre-vocational and early-employment needs of adolescents and young adults with long term health conditions from the perspectives of OTs and to examine their current and potential role in addressing these needs.

**Methods:**

A questionnaire was developed using Qualtrics software and piloted with two OTs and amended accordingly. A snowballing recruitment method via email and Twitter was employed. Initial contact was made via colleagues of the project team and cascaded to OT networks. Data included demographic, involvement in health care planning with education, perceived importance of addressing pre-vocational issues for young people, respondents’ knowledge and confidence, and the role of OT.

**Discussion/Results:**

The survey received 130 responses, of which 76 responses had complete data available (59%). A complete case analysis approach was taken. The majority of respondents were in clinical roles, primarily working in hospital or community settings and duration in practice ranged from 10 months to 40+ years. The perceived importance of addressing a wide range of vocational issues was consistently greater than the perceived knowledge and confidence of the professionals to address these. The majority of respondents (75%) perceived a need to address vocational issues for young people in their setting although only 22% reported that there was a designated member of their multidisciplinary team who would address these. The majority of such personnel identified by respondents were OTs but also included youth workers, education officer in the youth justice service, social worker.

63% routinely assessed productive/vocational occupations such as employment/education. Half of respondents (51%) did not know where to signpost/refer young people for vocational/employment advice. The majority (92%) reported not having any training in addressing vocational/employment issues specifically for young people aged 10-24 years and only 20% reported training in this area with adults. The majority (70%) however did perceive a difference in providing such advice to young people compared to adults. The main perceived barrier to addressing this area in practice was limited clinical time.

**Key learning points/Conclusion:**

This study echoes previous calls for vocational issues to be addressed with young people with long-term health conditions and highlights the need for this to be addressed in both training and service provision.

